# Naringenin Ultrafine Powder Was Prepared by a New Anti-Solvent Recrystallization Method

**DOI:** 10.3390/nano12122108

**Published:** 2022-06-19

**Authors:** Xiaonan Zhang, Yan Huang, Yufei Shi, Mengyu Chen, Lubin Zhang, Yimin An, Zhiwei Liu

**Affiliations:** 1 School of Life Science, Jiaying University, Meizhou 514015, China; hyanee@163.com (Y.H.); shishang812022@163.com (Y.S.); mengyuchen@163.com (M.C.); rubzhang@126.com (L.Z.); yiminan@jyu.edu.cn (Y.A.); 2 Guangdong Provincial Key Laboratory of Conservation and Precision Utilization of Characteristic Agricultural Resources in Mountainous Areas, Meizhou 514015, China

**Keywords:** naringenin, antisolvent recrystallization, powder properties, antioxidant activity

## Abstract

Raw naringenin directly isolated from plants is significantly limited by its poor dissolution rate and low bioavailability for clinical and in vivo studies. This study reported a method for the preparation of naringenin ultrafine powder (NUP) using a novel anti-solvent recrystallization process; preliminary experiments were conducted using six single-factor experiments. The response surface Box–Behnken (BBD) design was used to optimize the level of factors. The optimal preparation conditions of the DMP were obtained as follows: the feed rate was 40.82 mL/min, the solution concentration was 20.63 mg/mL, and the surfactant ratio was 0.62%. The minimum average particle size was 305.58 ± 0.37 nm in the derived optimum conditions. A scanning electron microscope was used to compare and analyze the appearance and morphology of the powder before and after preparation. The characterization results of FTIR, TG and XRD showed that no chemical change occurred in the powder before and after preparation. Through the simulated gastrointestinal juice digestion experiment, it was confirmed that the absorption rate of NUP was 2.96 times and 4.05 times higher than raw naringenin, respectively. Therefore, the results showed that the reduction in the particle size through the use of low-speed recrystallization could improve the absorption rate and provided a feasible approach for the further applications.

## 1. Introduction

Naringenin is a polymethoxyl flavonoid widely found in the peel of citrus plants, which has great significance for human health [1]. Naringenin is widely distributed in pomelo peel [2], grapefruits [3], bergamots and tomatoes [4] et al. In recent years, it has attracted extensive attention due to its clinical nature. Naringenin is a powder, white to light yellow in color [5]. Naringenin has good anti-inflammatory [6], antioxidant [7] and anti-tumor effects [8,9,10], and is also clinically used in the treatment of a variety of bacterial infections [11]. Naringenin is soluble in ethanol [12] and is often used in the health food and pharmaceutical fields [13]. However, raw naringenin directly isolated from plants exhibits a poor dissolution rate and low bioavailability, which greatly limits its clinical application [14]. Dong et al. reported that reducing the particle sizes of insoluble substances by increasing their specific surface area is an effective method for increasing dissolution rate [15]. The bioavailability of these substances is effectively increased by preparation with ultrafine technology [16].

Burra M. et al. enhanced the bioavailability of simulated intestinal tracts by studying lipid-based nanoscale raloxifene hydrochloride powder [17], and Tao Liu et al. studied the effects of nanoparticle sizes on the dissolution rates of powders [18]. The preparation of solid liposome nanoparticles resulted in better biocompatibility [19]. Conventional small molecule preparation methods include ultrasonic crushing [20], wet grinding [21], supercritical rapid expansion and ultrafine-powder prepared by high speed homogenate assisted anti-solvent method.The liquid antisolvent recrystallization method has the potential to be applied in the pharmaceutical industry, which allows for rapidity, convenience, low cost, and high yield preparation. The technique is based on the change from supersaturation caused by the mixing of miscible solvent and antisolvent, which has successfully been applied to isoflavone [16], daidzein [22], and so on. There are few studies on the antisolvent recrystallization under the low-speed (≤2000 rpm) homogenate process (ARLM). When the rotational speed of homogenizing slurry is lower than 2000 rpm, it has a good energy saving effect, and helps to reduce noise pollution and solve the splash phenomenon in the homogenizing slurry system [22]. Compared with the high-speed homogenization antisolvent recrystallization method, ARLM can reduce friction and the heat generated. ARLM has been successfully applied in the preparation of trans-cinnamic acid nanoparticles [23]. A low-temperature environment is conducive to the generation of small molecules, while a high temperature will lead to particle aggregation and eventually produces many powders with large particle sizes [24]. Therefore, low-speed homogenization is conducive to the generation of small particles.

Therefore, we used ARLM to prepare NUP and then collected the dried ultrafine powders. In conclusion, the preparation of naringenin nanoparticles by a low-speed homogenization method combined with an antisolvent has great research value.

## 2. Materials and Methods

### 2.1. Materials

**Figure 1:**
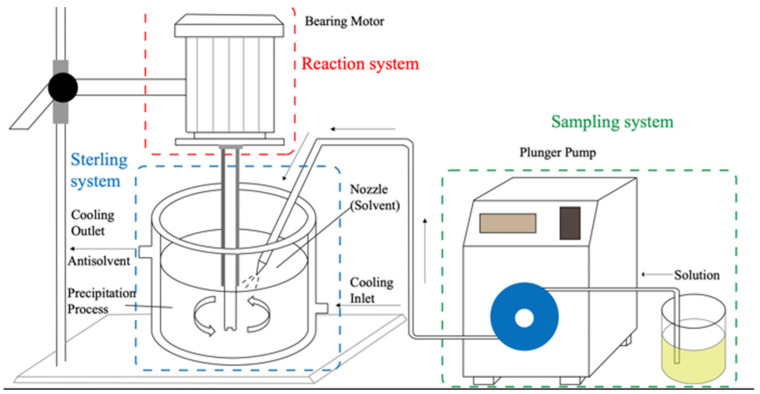
Antisolvent recrystallization under low-speed homogenate process (includes sampling system, reaction system and sterling system). Reprinted from ref. [22].

Naringenin (purity ≥ 98%), salicylic, disodium hydrogen phosphate, ferric chloride, trichloroacetic acid, and other items were purchased from Baodi chemical Co. Ltd. (Tianjin China). Simulated artificial gastric juice (AGJ) and artificial intestinal juice (AIJ) were purchased from Yuanye Biotechnology Co., Ltd. (Shanghai, China). DMSO and ethanol are analytical grade, purchased from Shanghai Yongfa Co., LTD. Other reagents were purchased from Baodi Co. Ltd (Tianjin, China). The spiral nozzle was purchased from Machinery Manufacturing Co., LTD (Shanghai, Jinke); the diameter of the nozzle was 200–600 μm. When efflux, due to the special structure of the spiral nozzle (Figure 1), the solution is scattered by the high pressure spiral, and the anti-solvent is evenly mixed, which contributes to the good dispersion and uniformity of the prepared particles.

### 2.2. Preparation Process

We prepare NUP using ARLM; the anti-solvent recrystallization unit consists of a sampling system, a reaction system, and a sterling system with a volume of 1000 mL and a low-speed homogenization section with a volume of 1000–3000 rpm, as well as a peristaltic pump with jet speeds ranging from 9 to 45 mL/min (Figure 1). The size range of the jet system is 200–600 μm. At the beginning of the experiment, the prepared naringenin solution was transported to the injection system through a vacuum peristaltic pump, and the solution was sprayed into a mist under pressure to form small droplets. Under the action of the ultrasonic wave and low speed homogenizing system (constant temperature 5 °C), the solution and antisolvent fully reflect and recrystallize into numerous tiny nano-crystalline nuclei.

### 2.3. Preparation of Naringenin Microparticles

NUP was prepared using the ARLM method. After the initial tests, a certain concentration of naringenin was dissolved by DMSO at room temperature, and the samples were injected into the anti-solvent (deionized water) in the reaction tank at a drop rate of 9–45 mL/min in ARLM. NUP filter cake was filtered (0.45 μm), and dried at 60 °C to obtain NUP powder. During the experiment, the average particle size distribution range of NUP was monitored by a dynamic light scatterer and the average particle size (APS) was calculated.

### 2.4. Experimental Optimization

The following six single factors were set as the initial results: X1 (percentage of surfactants), X2 (solution concentration), X3 (nozzle size), X4 (homogenization speed), X5 (liquid-liquid ratio) and X6 (feed speed). Through the above optimization, we obtained the best process conditions and minimum particle size value and drew a chart for the analysis and discussion (Table 1).

### 2.5. Determination of Powder Properties

#### 2.5.1. Scanning Electron Microscopy

The particle size and appearance of the naringenin and NUP samples were observed by SEM (JSM-7500, Japan Electronics Co., Ltd, Tokyo, Japan). The sample powder was dried and fixed on a tray with adhesive tape, and then a layer of conductive metal particles was sprayed on the surface of the powders. When the current was 50 mA, the sample appearance was analyzed for morphology and particle size.

#### 2.5.2. Fourier Transform Infrared Spectroscopy

We used infrared spectroscopy (Magna-IR560, Nicolet instrument corporation, Madison, WI, USA) to record the powder properties of raw dyewood and NUP samples. The samples were pretreated with 1% Kbr, pressed into thin slices and scanned in the wave-number range of 500–4500 cm^−1^ to record the characteristic peaks and analyze the results.

#### 2.5.3. X-ray

The crystal morphology of the naringenin samples was analyzed by an XRD powder diffractometer (Philips, Xpert-Pro. PANalytical BV, Almelo, The Netherlands). The dye lignin small particle sample (10 mg) was dispersed evenly on the glass slide for further use. Irradiation was carried out through Cu Ka1 at 30 mA and 40 kV. The XRD diffraction spectrum was 2 h. The scanning rate was 5°/min, the scanning range was 10~70° and the step length was 0.02°.

#### 2.5.4. DSC

A thermogravimetric analyzer (TA instruments, model, DSC 204, Woodland, CA, USA) was used to analyze the naringenin samples, and the conditions were as follows: 3 mg samples were placed in an open aluminum pot. The experimental heating rate was 10 °C/min, and the nitrogen flow was 50 mL/min. The weight loss percentage of samples was monitored between 25 and 350 °C.

### 2.6. Dissolution Rate Analysis

An amount of 5 mg of naringenin samples were dispersed in 10 mL of AIJ and AGJ, respectively. After full stirring, the samples were centrifuged at 12,000 rpm for 10 min, filtered at 0.45 μm, and transferred to a dialysis membrane bag (molecular retention value of 3500). The samples were further stirred and released in 500 mL of AGJ and AIJ. The experimental reaction was carried out at 37℃. At a fixed time (5, 15, 30, 45, 60, 75, 90 and 120 min), 0.2 mL samples were taken from each index at 8 different time points; the HPLC method was used to measure the dissolution rate of the samples and to draw the curve. The HPLC conditions were as follows: C18 column (4.6 × 100 mm^2^, 5 μm, Agilent LTD, Palo Alto, CA, USA). The mobile phase included methanol, which was mixed with phosphoric acid aqueous solution (50:50) at a flow rate of 1 mL/min with a volume fraction of 0.1%. The detection wavelength was 250 nm and the running time was 15 min. The experiment was carried out three times.

### 2.7. Solvent Residue Analysis

The residual solvent in NUP was analyzed by a gas chromatograph equipped with a flame ionization detector (Agilent G1540N-210, Palo Alto, CA, USA). NUP methanol solution was configured with 10 mg/mL, centrifuged at 10,000 rpm for 10 min, and the supernatant was filtered and extracted. The detection conditions were as follows: the detector operated at 280 °C, the flow rate of nitrogen was 2.2 mL/min, and the detector was retained for 5 min. The injection volume was 10 μL.

## 3. Results and Discussion

### 3.1. Discussion of the Results of Each Factor

In this study, we summarize the effects of various factors on NUP size. With the other factors constant (surfactant concentration of 0.6%, solution concentration of 15 mg/mL, nozzle size of 400 μm, homogenization speed of 2000 rpm, anti-solvent solvent ratio of 9, and feed rate of 27 mL/min), the effects of various factors and levels on particle size were investigated respectively.

**Figure 2:**
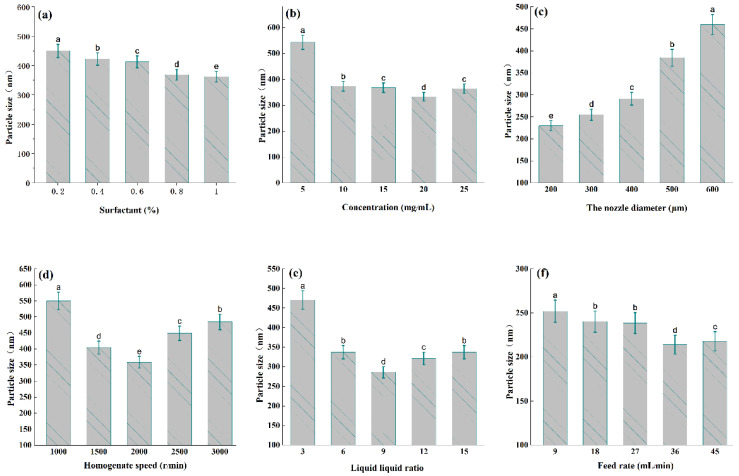
Influence of various factors on NUP size ((**a**): surfactant %, (**b**): concentration mg/mL, (**c**): nozzle diameter μm, (**d**): homogenizer speed r/min, (**e**): liquid-liquid ratio, (**f**): feed rate mL/min). The different letters (a–e) indicate significant differences among all samples (*p* < 0.05).

As is shown in Figure 2a, the surfactant can bind to the surface of ultrafine powder to prevent the agglomeration and growth of ultrafine particles. We investigated the effect on NUP by adding different concentrations (0.2–1%) of twain 80. As can be observed from Figure 2a, when the additive amount of surfactant reached 1%, the NUP particle size decreased to 362.23 nm; therefore, 1% twain concentration was selected as the additive amount for the next study.

Figure 2b shows the particle size range of 325.32–549.10 nm with different concentrations (5, 10, 15, 20 and 25 mg/mL). When the concentration of naringenin is 20 mg/mL, Figure 2b shows the particle size range of 325.32–549.10 nm of NUP. The above phenomenon may be attributed to the fact that the low concentration (5–20 mg/mL) system viscosity is low, the recrystallization phenomenon in the reaction tank is uniform, and the adhesion phenomenon does not easily occur under the action of homogenate, so it is beneficial to reduce the diameter of the crystal nucleus. When the solution concentration increases, the collision of small molecules in the solution system increases, and the particle size may agglomerate instead, resulting in larger particle sizes in the deposition process.

It is evident that the smaller the size of the jet hole, the smaller the particle size, which is a rule, but in the process of particle preparation, the jet hole with a small particle size is easy to plug in this process, resulting in increased management pressure, which results in liquid splashing (Figure 2c). When the jet hole is >500 μm, the particle size increases greatly and reaches 460 nm, correspondingly. This situation may be caused by the decrease in pressure in the pipeline, which is consistent with the study of Yu et al. [25].

The effects of the low-speed homogenate (1000–3000 rpm) on the average grain size of NUP were studied. As shown in Figure 2d, the minimum particle size (363.38 nm) was obtained when the optimal stirring speed was 2000 rpm. The solution tends to be stable in the process of jet flow (crystal cannot agglomerate under stable growth), but with the increase in stirring speed, the equilibrium of the stable state is destroyed, so the generated particle size gradually becomes larger. In addition, studies have shown that a large amount of heat will be generated in the process of high-speed homogenization (≥5000 rpm); as the system velocity continues to increase, the equilibrium of the stable state of the high-speed solution system is destroyed, meaning that the recrystallized particle size gradually increases [23].

According to Figure 2e, when the liquid-liquid ratio is 9, NUP has a minimum particle size of 280 nm. It can be inferred that when the ratio is 9, the system in the reaction process is in a stable state, and recrystallization does not easily agglomerate in this state, which is consistent with the work of Lacerda et al. [26]. When the feed rate range is 36–45 mL/min, the particle size of NUP decreases (Figure 2f); this may be because the anti-solvent system is in a stable state when the sample is at 36 mL/min, and the nucleation rate of the recrystallized particles does not further aggregate. A smaller particle size range is generated and tends to be stable [26].

### 3.2. Optimization of Particle Size by Response Surface Methodology (RSM)

In order to further verify the above results, we used *RSM* to optimize the parameters. *RSM* investigated and optimized the interaction between *X*_1_ (percentage of surfactants), *X*_2_ (solution concentration) and *X*_3_ (feed speed). *Y* (*APS)* was taken as the optimized operating condition. The experimental design and results are shown in Table 2. The quadratic model was chosen as the statistical model of least square response optimization. The following parameters were used:

(1) $$Y=306.46-16.09\;X_{1}-17.01\;X_{2}-5.84\;X_{3}-2.18\;\;X_{1}\;X_{2}-1.35\;\;X_{1}X_{3}-1.29X_{2}X_{3}+16.01X_{1}^{2}+42.39\;X_{2}^{2}+28.18\;X_{3}^{2}$$

A total of 17 results were optimized by design-Expert 10. The results of the fitted regression model (*p* < 0.0001) show that the results of the equation are extremely significant; the predicted *R*^2^ was 0.9969, so it is reasonable to use this equation to simulate the relationship between the index values and factors. The experimental range of the *APS* results affected by the independent variables is shown in Table 2.

**Figure 3:**
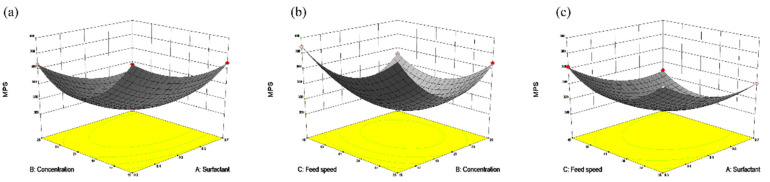
Response surface optimization of NUP particle size (optimization of concentration, surfactant and feed speed factors and levels). (**a**): Effect of concentration and surfactant on particle size, (**b**): The effects of feed speed and concentration on particle size, and (**c**): The effects of feed speed and surfactant on particle size.

As the concentration of the naringenin and the surfactant gradually increased (Figure 3a), the *APS* of the NUP tended to decrease first and then increased. As is shown in F Figure 3b, with the slight increase in feeding speed and concentration, NUP gradually decreases first and then increases, which may be due to the high concentration (21–25 mg/mL), leading to the adhesion of particles in the jet process, which is not conducive to the formation of small particles. Meanwhile, too fast feeding speed (41–45 mL/min) will lead to the increase in flow per unit time, which is not conducive to the formation of small particles [14].

As shown in Figure 3c, NUP gradually decreases and remains flat with the increase in the surfactant and feeding speed. The surfactant can effectively promote the dispersion of NUP in the solution system and form a uniform anti-solvent system that is conducive to the formation of small crystals in this process, which is consistent with the law studied by Akhter et al. [27].

The above studies show that when the ARLM process was combined with the Box–Behnken analysis, the optimal concentration was 20.63 mg/mL, the proportion of surfactant was 0.62% and the feed speed was 40.82 mL/min. The minimum particle size of NUP was 305.58 ± 0.37 nm, which is almost consistent with the expected value of 306.46 ± 0.29 nm obtained from the model equation, indicating that the response model analysis accurately reflected the results of the experimental optimization.

### 3.3. Powder Properties

#### 3.3.1. Microscopic Morphology of NUP

**Figure 4:**
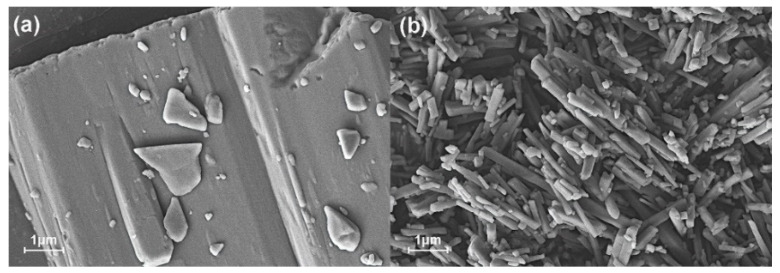
The morphology of raw naringenin (**a**) and NUP (**b**) under a scanning electron microscope.

We used SEM to study the appearance and morphology of the raw naringenin and NUP powder (Figure 4a). The observation showed that the raw naringin powder has a large particle size, which is massive powder, and the irregular particle shape might be the main reason for the poor dissolution rate of the naringenin powder. The particle size of NUP was small (Figure 4b), the powder particles were relatively uniform and small, with an average diameter of less than 500 nm. After preparation, the specific surface area of the ultrafine powder increased with good dispersibility. The particle size of the NUP powder prepared by the ARLM method was smaller than that of raw naringenin, and the uniformity of the NUP powder was better. It is also speculated that the dissolution rate of NUP is better than that of raw naringenin [14].

#### 3.3.2. XRD Analysis

**Figure 5:**
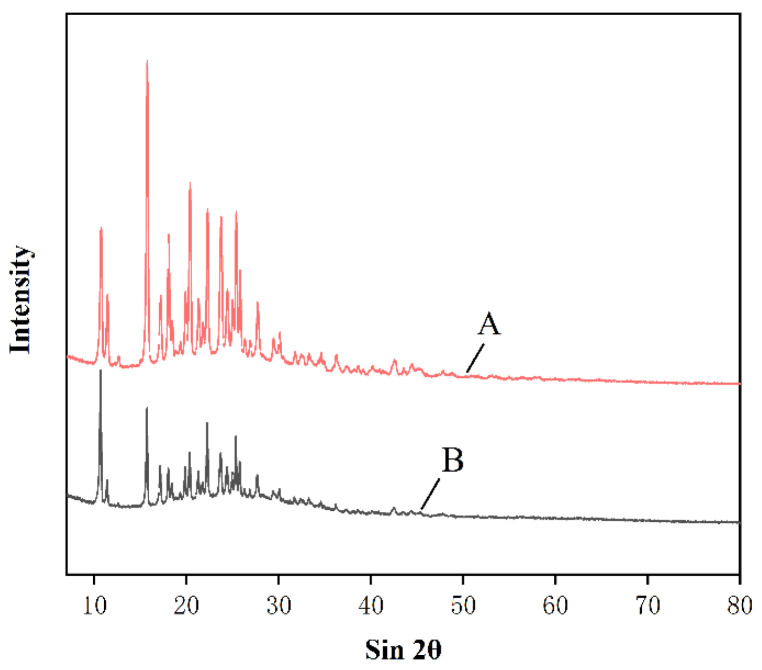
Comparison of raw naringenin (**A**) and NUP (**B**) under X-ray diffraction conditions.

As is shown in Figure 5, raw naringenin has obvious spikes at the diffraction angles of 32.17°–49.09°, indicating that the drug is crystalline. The overall peak height of NUP is weak and small, the size of the diffraction peak is due to the decrease in particle crystalline or particle size. This indicates that NUP after ARLM treatment is small in size and has poor crystallinity. Drugs with lower crystallinity have faster dissolution rates and bioavailability [28].

#### 3.3.3. FTIR Analysis

**Figure 6:**
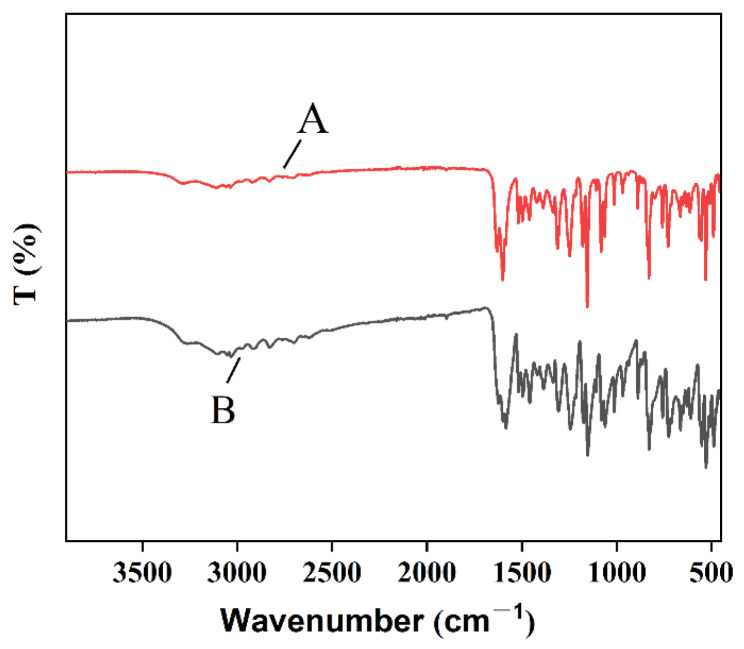
Comparison of raw naringenin (**A**) and NUP (**B**) under FTIR conditions.

The comparison of infrared absorption peaks of the naringenin samples was carried out. By comparing the absorption intensity of the spectral bands and the content of the chemical groups, it can be observed that the peak positions, peak shapes and trends of the two samples are basically the same (Figure 6), indicating that the properties of the naringenin powder have not changed before and after preparation. Therefore, the spectra before and after the preparation of naringenin have the same vibration frequency, and the chemical structure has no changes [29].

#### 3.3.4. Thermodynamic (TG)

**Figure 7:**
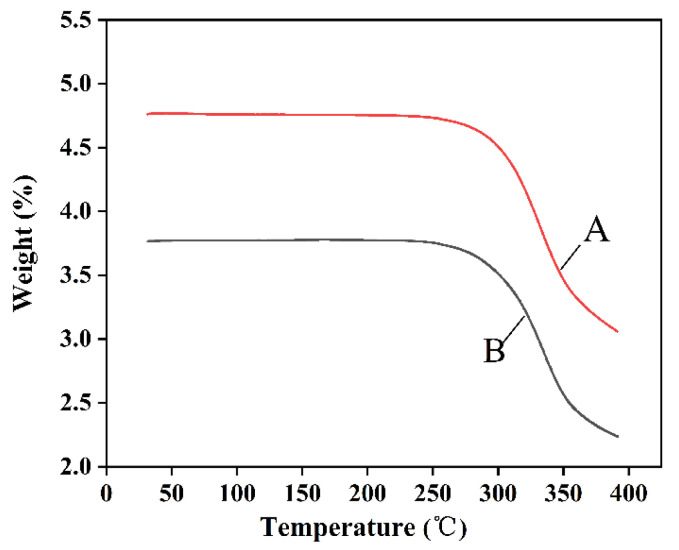
DSC curves of raw naringenin (**A**) and NUP (**B**).

As is shown in the Figure 7, the energy absorption trends of the two samples are essentially the same. With the increasing temperature, the naringenin samples volatilized gradually with the same trend. The raw naringenin and NUP curves are basically parallel, indicating that the composition of the samples did not change before and after preparation.

### 3.4. Dissolution Rate Analysis

**Figure 8:**
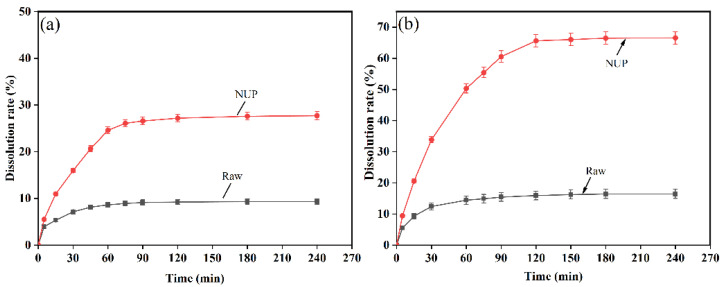
Cumulative release of the naringenin simples in (**a**) the simulated gastric fluid and (**b**) the simulated intestinal fluid.

Figure 8 shows the dissolution rate analysis of naringenin before and after preparation. According to the standard curve regression equation (*Y =* 63.23*x* − 17.35, *R*^2^ *=* 0.9996), after long-term metabolism and dissolution at 37 °C, the dissolution rate of NUP in AIJ was 7.43 ± 0.11 mg/mL and that in AGJ was 18.18 ± 0.21 mg/mL. The dissolution rate of the naringenin powder in AIJ and AGJ was 2.52 and 4.49 mg/mL, respectively. According to the above data, at 37 °C, the dissolution rate of raw naringenin powder is 2.96 times that of NUP in AIJ and 4.05 times in AGJ. The dissolution rates of naringenin in AGJ and AIJ were 9.34% and 27.70%, 16.43% and 66.56%, respectively. According to the Oswald–Freundlich equation, when the system is stable, the smaller crystal or sol particles in the solute will be better dispersed to maximize the specific surface area, and the smaller particle size is conducive to increasing the dissolution rate. Therefore, the dissolution rate of NUP prepared by the above method is greatly enhanced [30].

## 4. Conclusions

Taking the particle size of naringenin as the main index, we optimized it using the BBD method, and obtained NUP with a minimum particle size of 305.58 ± 0.37 nm. The optimized conditions are as follows: the optimal concentration is 20.63 mg/mL, the dosage of surfactant is 0.62%, and the feed rate is 40.82 mL/min. In addition, the powder before and after preparation was characterized by XRD, DSC and FTIR and the properties of the powder did not change. However, the dissolution rate of NUP in AIJ and AGJ increased by 2.96 times and by 4.05 times, respectively. Through the use of the ARLM process, we were able to obtain a high-performance NUP powder, and provided technical support for further application of naringenin functional properties.

## Figures and Tables

**Table 1:** Influencing factors and the corresponding levels (L).

	Factors	Unit	L1	L2	L3	L4	L5
X1	Percentage of surfactants	%	0.2	0.4	0.6	0.8	1
X2	Solution concentration	mg/mL	5	10	15	20	25
X3	Nozzle size	μm	200	300	400	500	600
X4	Homogenization speed	r/min	1000	1500	2000	2500	3000
X5	Liquid-liquid ratio	mL/mL	3	6	9	12	15
X6	Feed speed	mL/min	9	18	27	36	45

**Table 2:** Box–Behnken design (BBD) with the experimental value for NUP size (nm), analysis of variance (ANOVA) for the response surface quadratic model, and fit statistics for the response values.

No	BBD Experiments				ANOVA							
	*X*_1_	*X*_2_	*X*_3_	*Y*	Source	Sum of Squares	Degree of Freedom			Mean Square	F-Value	*p*-Value
1	0.3	15	40	307.03	Model	17,849.04	9			1983.23	2850.28	<0.0001 **
2	0.7	15	40	388.68	*X*_1_	2071.75	1			2071.75	2977.51	<0.0001 **
3	0.3	25	40	329.38	*X*_2_	2315.40	1			2315.40	3327.68	<0.0001 **
4	0.7	25	40	395.99	*X*_3_	272.61	1			272.61	391.80	<0.0001 **
5	0.3	20	35	370.65	*X*_1 × 2_	19.98	1			19.05	27.38	0.0012
6	0.7	20	35	307.11	*X*_1 × 3_	7.32	1			7.32	10.52	0.0142
7	0.3	20	45	305.69	*X*_2_ *X*_3_	6.68	1			6.68	9.60	0.0173
8	0.7	20	45	368.21	*X*_1_^2^	1073	1			1079.75	1551.81	<0.0001 **
9	0.5	15	35	362.89	*X*_2_^2^	7551	1			7567.28	10,875.66	<0.0001 **
10	0.5	25	35	305.97	*X*_3_^2^	3333	1			3344.52	4806.73	<0.0001 **
11	0.5	15	45	365.89	Residual	4.81	7			0.70		
12	0.5	25	45	367.98	Lack of fit	3.32	3			1.11	2.85	0.1688
13	0.5	20	40	306.53	Pure error	1.55	4			0.39		
14	0.5	20	40	398.98	Corrected total	17,853.91	16					
15	0.5	20	40	352.51	Credibility analysis of the regression equations							
16	0.5	20	40	327.96	*S.D.*	*Mean*	*CV (%)*	*R* ^2^	*Adj. R* ^2^	*Pre. R^2^*		*Ade. Pre.*
17	0.5	20	40	341.13	0.83	347.21	0.24	0.9997	0.9994	0.9969		144.015

*X*_1_: Percentage of surfactants; *X*_2_: solution concentration; *X*_3_: feed speed. *Y*: APS. “**”: extremely Significant.

## Data Availability

The data presented in this study are available on request from the corresponding author.
